# Association of different types of liver disease with demographic and clinical factors

**DOI:** 10.7603/s40681-016-0016-2

**Published:** 2016-08-13

**Authors:** Kao-Chi Cheng, Wen-Yuan Lin, Chiu-Shong Liu, Cheng-Chieh Lin, Hsueh-Chou Lai, Shih-Wei Lai

**Affiliations:** 1 College of Medicine, China Medical University, No. 2, Yuh-Der Road, 404 Taichung, Taiwan; 2 Department of Family Medicine, China Medical University Hospital, 404 Taichung, Taiwan; 3 School of Chinese Medicine, China Medical University, 404 Taichung, Taiwan; 4 Department of Internal Medicine, China Medical University Hospital, 404 Taichung, Taiwan

**Keywords:** Fatty liver, Globulin-albumin ratio, Hepatitis, Cirrhosis, Liver cyst, Hemangioma

## Abstract

**Background and Aim:**

A metric that predicts the presence of cancer-related liver disease would allow early implementation of treatment. We compared the demographic and clinical characteristics of patients with no evidence of liver disease, with a cancer-associated liver disease, and with a liver disease not associated with cancer.

**Methods:**

Retrospective, hospital-based, cross-sectional study which reviewed the medical records of subjects who underwent health examinations at a Taiwanese hospital from 2000 to 2004 and who had normal levels of amino transaminases. Demographic and clinical data were analyzed by univariate and multivariate statistics.

**Results:**

A total of 2344 subjects had no evidence of liver disease (non-LD), and 1918 subjects had at least one liver disease (LD). The LD group was further divided into those with a cancer-associated liver disease (LD-1, n = 1632) and those with a liver disease not associated with cancer (LD-2, n = 286). Age, BMI, percentage of males, globulin:albumin ratio (G/A), percentage of patients with gallstones, AST, and ALT were significantly higher in the LD group. Univariate analysis showed that the G/A was significantly higher in the LD-2 group than the LD-1 group; multivariate analysis indicated that the G/A was not independently associated with liver disease, but that subjects who were older and had higher BMI were significantly more likely to have a cancer-associated liver disease. Conclusions: For patients with liver disease, a multivariate model can be used to distinguish those with a cancer-associated liver disease from those with a liver disease not associated with cancer.

## 1. Introduction

Liver disease includes many diverse conditions, diseases, and infections that affect the morphology and function of the liver [1]. Alterations in the liver function test (LFT) and jaundice are typical and easily observable manifestations of liver disease [1]. Some liver diseases are associated with increased risk for cancer, including cirrhosis, in which the development of fibrotic tissues and scars leads to reduced liver function; fatty liver disease (FLD), which often occurs in patients with the metabolic syndrome and/ or those who consume excessive alcohol; and hepatoma, a tumor that is typically cancerous. Other forms of liver disease are not associated with cancer, including hepatic cysts, which may be congenital or pathogen-associated; and benign neoplasia, such as hemangiomas or adenomas [1].

Traditionally, definitive diagnosis of a liver disease requires analysis of biopsy specimens and/or imaging data provided by ultrasonography, laparoscopy, or computed tomography. Liver biopsy is the gold standard for diagnosis of most liver diseases, but this must be performed by an expert pathologist, is costly, and is associated with potentially serious adverse effects, such as intraperitoneal hemorrhage [2]. Thus, biopsy is often considered unnecessary for diagnosis if adequate laboratory, clinical, and imaging data are available.

Several reports have suggested that cirrhosis may be present if the aspartate transaminase: alanine transaminase ratio (AST/ALT) is greater than 1.0 [3, 4]. A previous study also showed that the serum globulin/albumin ratio (G/A) is elevated in hepatitis C patients with liver cirrhosis [5]. Clearly, a simple metric that could predict cancer-associated liver disease would have great clinical importance, because this would allow early implementation of treatment.

**Fig. 1 -:**
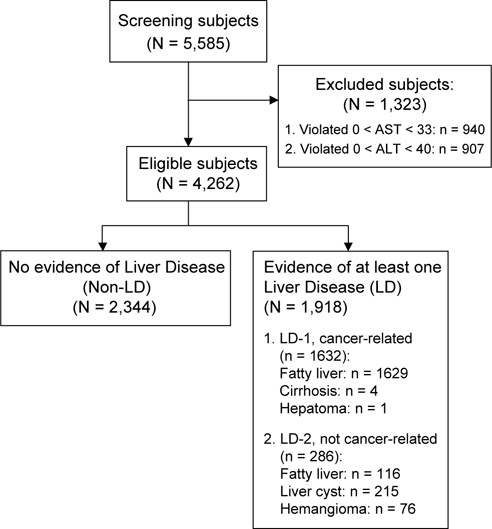
**Patient disposition**

In this retrospective study, we compared numerous demographic and clinical factors of patients with a cancer-associated liver disease and patients with liver diseases that are not associated with cancer.

## 2. Materials and methods

### 2.1. Study population

This was a retrospective, hospital-based, cross-sectional study in which the medical records of 5,585 subjects who underwent health examinations at the China Medical University Hospital (Taichung, Taiwan) from 2000 to 2004 were screened (Figure 1). All 4,262 eligible subjects had normal levels of serum aminotransferases, based on standards established by the Chinese Christian Hospital (AST < 33 U/l, ALT < 40 U/l). The institutional review board of the China Medical University Hospital approved this research.

### 2.2. Data collection and diagnostic criteria

Subjects who never drank alcohol were classified as “non-drinkers”; those who reported drinking alcohol often were classified as “habitual drinkers”. Fatty liver disease, cirrhosis, gallstones, and splenomegaly were all diagnosed by abdominal sonography[6]. Fatty liver disease was diagnosed if the liver had homogenously increased echogenicity and a smooth surface. Cirrhosis was diagnosed if there was increased parenchymal echogenicity, poor tissue penetration, and parenchymal inhomogeneity. Liver cysts and hemangiomas were diagnosed based on their characteristic sonographic signals. Gallstones were diagnosed based on a characteristic increased echogenicity anywhere in the biliary tree, and included patients who had “silent stones”. Splenomegaly was diagnosed if the spleen was greater than 12 cm in length and had a maximal transverse diameter of 5 cm.

All venous blood samples were obtained in the morning after a 12 h overnight fast. Globulin, albumin, and other serum parameters were analyzed by a biochemical autoanalyser (Hitachi 736-15, Tokyo, Japan) at the Department of Clinical Laboratory within 4 h of collection. Hepatitis B surface antigen was detected by an ELISA test from Enzygnost, Dade Behring GmbH (Marburg, Germany) and the antibody to hepatitis C virus was detected by an ELISA test from Abbott (HCV EIA 3.0, Abbott Laboratories. Abbott Park, Illinois).

### 2.3. Statistical analysis

Data are expressed as means and standard deviations for age, body mass index (BMI), AST, and ALT, and as number and percentage for other characteristics. All subjects were classified as having no evidence of liver disease (non-LD), a liver disease that was associated with cancer (LD-1, including cancer-related FLD, cirrhosis, or hepatoma), or a liver disease not associated with cancer (LD-2, including cancer-unrelated FLD, cyst or hemangioma) based on symptoms and ultrasonography. Inter-group comparisons were performed with Pearson’s *Chi*-square test, a one-way ANOVA, or a two sample *t*-test. A simple linear regression model was used to identify the correlation of the G/A with specific patient characteristics. Then, a multivariate logistic regression model was used to predict the probability of a subject being diagnosed with liver disease relative to the G/A and the significant characteristics identified by univariate analysis. For all statistical tests, a *P* value < 0.05 was considered significant. All data were analyzed using SAS 9.0 (SAS Institute Inc., Cary, NC, USA).

## 3. Results

Figure 1 shows the patient selection protocol. From 2000 to 2004, we screened 5,585 subjects initially, and excluded 940 subjects due to elevated AST (> 33 U/l) and 907 subjects due to elevated ALT (> 40 U/l). Among the 4,262 eligible subjects, 2,344 subjects had no evidence of liver disease (non-LD group) and 1918 subjects had at least one liver disease (LD group). In the LD group, we classified subjects as having a liver disease associated with cancer (LD-1, n = 1632), which includes those with cancer-associated fatty livers, cirrhosis, or hepatoma, or as having a liver disease not associated with cancer (LD-2, n = 286), which includes those with fatty livers not associated with cancer, liver cysts, or hemangiomas.

Table 1 shows the basic demographic and clinical characteristics of the non-LD and LD groups. Among demographic characteristics, age, BMI, and percentage of males were significantly higher in the LD group. Among the clinical characteristics, the G/A, percentage of patients with gallstones, level of AST, and level of ALT were significantly higher in the LD group.

Table 2 shows the demographic and clinical characteristics of the non-LD, LD-1, and LD-2 groups. The characteristics of these three groups were compared with a one-way ANOVA or Pearson’s *Chi*-square test. The results indicate significant differences in these three groups for age, BMI, sex, alcohol usage, G/A ratio, AST, ALT, and heptatis C virus (HCV).

A previous study [5] indicated that HCV patients with G/A ratios greater than 1 had a high probability of cirrhosis, with an odds ratio (OR) of 31.47 (*P* = 0.008). Thus, we performed a simple linear regression model of G/A ratio and patient characteristics for all 4262 subjects (Table 3). The results show that the G/A was significantly higher in subjects with a liver disease not associated with cancer (LD-2), but was not significantly higher in subjects with a liver disease associated with cancer (LD-1). When LD-1 and LD-2 were pooled, the G/A was significantly higher than non-LD patients. Increased age, male sex, and higher BMI were associated with a high G/A; the G/A was significantly lower for drinkers, and subjects with low ALT.

**Table 1:** 

Variables	Total (N = 4,262)	non-LD (n = 2,344)	LD (n = 1,918)	P value
Age^1^, years	49.1 (12.4)	47.0 (12.8)	51.8 (11.3)	< .001*
Sex^2^, males (%)	2,172 (51.0)	1,059 (45.2)	1,113 (58.0)	< .001*
BMI^1^, Kg/m^2^	23.7 (3.4)	22.4 (3.0)	25.3 (3.3)	< .001*
Alcohol usage^2^, drinkers (%)	387 (9.1)	211 (9.0)	176 (9.2)	0.844
Fatty liver^2^, n (%)	1,745 (40.9)	0 (0)	1,745 (91.0)	< .001*
Liver cyst^2^, n (%)	218 (5.1)	0 (0)	215 (11.2)	< .001*
Hemangioma^2^, n (%)	76 (1.8)	0 (0)	76 (4.0)	< .001*
Liver cirrhosis^2^, n (%)	4 (0.1)	0 (0)	4 (0.2)	0.027*
Hepatoma^2^, n (%)	1 (0.02)	0 (0)	1 (0.05)	0.269
Splenomegaly^2^, n (%)	50 (1.2)	27 (1.2)	23 (1.2)	0.887
Gallstones^2^, n (%)	210 (4.9)	93 (4.0)	117 (6.1)	< .001*
G/A ratio^2^, %	74.32 (14.59)	73.79 (14.83)	74.97 (14.27)	0.009*
AST^1^	23.0 (4.2)	22.5 (4.2)	23.6 (4.0)	< .001*
ALT^1^	21.0 (6.9)	19.3 (6.3)	23.1 (6.9)	< .001*
HBsAg^2^				
Positive	462 (12.6)	253 (12.9)	209 (12.3)	0.590
Negative	3,199 (87.4)	1,709 (87.1)	1,490 (87.7)	
Anti-HCV^2^				
Positive	47 (1.1)	29 (1.2)	18 (0.9)	0.362
Negative	4180 (98.9)	2,301 (98.8)	1,879 (99.1)	

Abbreviation: LD means subjects with liver disease liver diseases. Data were compared using [1] two sample *t*-test and [2] Pearson *Chi*-square test. * *P* value < 0.05.

**Table 2:** 

Variables	non-LD (n = 2,344)	LD-1 (n = 1632)	LD-2 (n = 286)	P value
Age^1^, years	47.0 (12.8)	51.4 (11.1)	54.1 (12.0)	< .001*
Sex^2^, males (%)	1,059 (45.2)	977 (59.9)	136 (47.5)	< .001*
BMI^1^, Kg/m^2^	22.4 (3.0)	25.6 (3.2)	23.5 (3.2)	< .001*
Alcohol usage^2^, drinkers (%)	211 (9.0)	163 (10.0)	13 (4.6)	0.013*
Fatty liver^2^, n (%)	0 (0)	1629 (99.8)	116 (40.6)	< .001*
Liver cyst^2^, n (%)	0 (0)	0 (0)	215 (75.2)	< .001*
Hemangioma^2^, n (%)	0 (0)	0 (0)	76 (26.6)	< .001*
Liver cirrhosis^2^, n (%)	0 (0)	4 (0.2)	0 (0)	0.061
Hepatoma^2^, n (%)	0 (0)	1 (0.06)	0 (0)	0.450
Splenomegaly^2^, n (%)	27 (1.2)	21 (1.3)	2 (0.7)	0.689
Gallstones^2^, n (%)	93 (4.0)	100 (6.1)	17 (5.9)	0.029*
G/A ratio^2^, %	73.79 (14.83)	74.5 (12.6)	77.7 (21.3)	< .001*
AST^1^	22.5 (4.2)	23.7 (4.0)	23.0 (4.2)	< .001*
ALT^1^	19.3 (6.3)	23.5 (6.9)	20.8 (6.9)	<.001*
HBsAg^2^				
Positive	253 (12.9)	177 (12.3)	32 (12.3)	0.864
Negative	1,709 (87.1)	1261 (87.7)	229 (87.7)	
Anti-HCV^2^				
Positive	29 (1.2)	11 (0.7)	7 (2.5)	0.019*
Negative	2,301 (98.8)	1604 (99.3)	275 (97.5)	

Abbreviations: LD-1: patients with cancer-related fatty liver, cirrhosis, or hepatoma; LD-2: patients with fatty liver, renal cyst, or hemangioma. Data were compared using [1] one-way ANOVA test, and [2] Pearson *Chi*-square test. * *P* value < 0.05.

**Table 3:** 

Variables	*β* (Std.err)	*P* value
**LD** ***vs.*** **non-LD (Reference)**	1.18 (0.45)	0.009*
**LD** ***vs.*** **Non-LD**		< .001*
Non-LD	Reference	-
LD-sub1	0.70 (0.47)	0.133
LD-sub2	3.90 (0.91)	< .001*
**Age, years**	0.27 (0.02)	< .001*
**Sex**		
Males	5.99 (0.44)	< .001*
Females	Reference	
**BMI, kg/m** ^2^	0.19 (0.06)	0.003*
**Alcohol usage,**		
Drinkers	-2.76 (0.78)	< .001*
Non-Drinkers	Reference	
**Splenomegaly**		
Yes	-3.82 (2.07)	0.066
No	Reference	
**Gallstones**		
Yes	1.54 (1.03)	0.134
No	Reference	
**AST**	0.06 (0.05)	0.252
**ALT**	-0.13 (0.03)	< .001*
**HBsAg**		
Positive	-0.79 (0.70)	0.258
Negative	Reference	
**Anti-HCV**		
Positive	3.71 (2.13)	0.082
Negative	Reference	

Abbreviations: LD-1: patients with cancer-associated liver disease; LD-2: patients liver disase not associated with cancer. *P value < 0.05

Table 4 shows the results of a simple linear regression model of G/A ratio and characteristics of patients in the LD-1 and LD-2 groups. Based on these results, subjects who were older, male, had higher BMI, and lower ALT had greater probability for a high G/A ratio.

Table 5 summarizes the results of univariate and multivariate logistic regression models of the risk for a cancer-associated liver disease (LD-1) relative to a liver disease not associated with cancer (LD-2) from all patients with signs or symptoms of liver disease (N = 1918). The univariate model indicated that G/A ratio, age, sex, BMI, AST and ALT all had significant effects on the risk of LD-2. However, the multivariate analysis indicates that only age and BMI had a significant effect on the risk of LD-2.

**Fig. 2 -:**
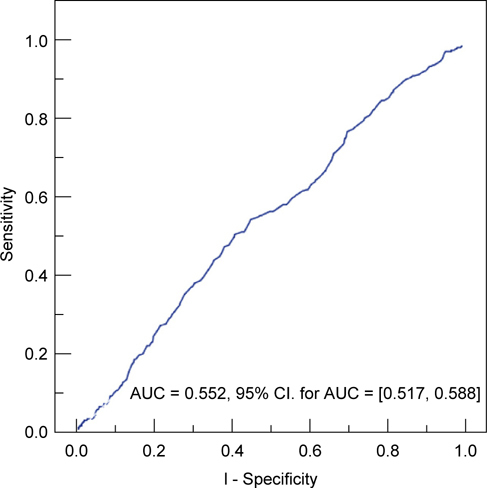
Predicted area under ROC curve (AUC) for the univariate model, which only considered G/A as an indicator of liver disease associated with cancer (LD-1) vs. liver disease not associated with cancer (LD-2).

Figure 2 shows the predicted area under the receiver operating characteristic (ROC) curve for a model that only considered G/A as an indicator of cancer-associated liver disease (LD-1). The area under the curve (AUC) was 0.552 (95% CI: 0.517-0.588). Thus, it is difficult to define an optimal cut-off point for G/A based on the univariate model.

**Fig. 3 -:**
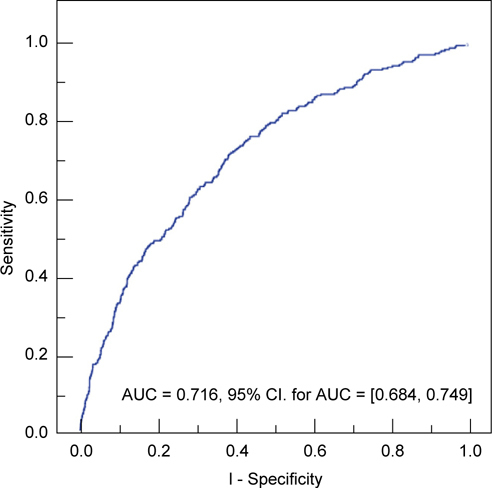
**Predicted area under ROC curve (AUC) for the multivariate model, which considered other confounding factors as indicators of liver disease associated with cancer (LD-1)** ***vs.*** **liver disease not associated with cancer (LD-2).**

Figure 3 shows the predicted area under the ROC curve for the multivariate model, which considered confounding factors (Table 5). The AUC was 0.716 (95% CI: 0.684-0.749). Clearly, the multivariate model is much better in distinguishing patients with cancer-associated liver disease (LD-1) from patients with a liver disease not associated with cancer (LD-2).

## 4. Discussion

Biopsy is considered the gold standard for the diagnosis of liver disease and cirrhosis, but is an invasive procedure associated with numerous limitations and complications. Biopsy can be expensive [7], is associated with possibly serious side effects [8], including potentially lethal intraperitoneal hemorrhage [2], and there can be significant sampling error between and within physicians [9]. Clearly, there is a need for a simple noninvasive clinical test or procedure that can predict different types of liver disease without biopsy.

**Table 4:** 

Variables	*β* (Std.err)	*P* value
**LD levels**		
LD-sub1	-3.20 (0.91)	< .001*
LD-sub2	Reference	
**Age, years**	0.29 (0.03)	< .001*
**Sex**		
Males	6.88 (0.64)	< .001*
Females	Reference	
**BMI, kg/m** ^2^	0.03 (0.10)	0.742
**Alcohol usage,**		
Drinkers	2.18 (1.13)	0.053
Non-Drinkers	Reference	
**Splenomegaly**		
Yes	5.25 (2.99)	0.079
No	Reference	
**Gallstones**		
Yes	-2.10 (1.36)	0.123
No	Reference	
**AST**	-0.06 (0.08)	0.487
**ALT**	-0.26 (0.05)	< .001*
**HBsAg**		
Positive	-0.79 (0.95)	0.401
Negative	Reference	
**Anti-HCV**		
Positive	2.31 (3.36)	0.491
Negative	Reference	

Abbreviations: LD-1: patients with cancer-associated liver disease; LD-2: patients liver disase not associated with cancer. *P value < 0.05

**Table 5:** 

	Univariate		Multivariate	
Variables	OR(95% CI. for OR)	*P* value	OR (95% CI. for OR)	*P* value
G/A ratio	1.01 (1.00-1.02)	0.002*	1.01 (0.99-1.02)	0.108
Age	1.02 (1.01-1.03)	< .001*	1.02 (1.01-1.03)	< .001*
Sex				
Male	0.61 (0.47-0.78)	< .001*	0.79 (0.60-1.05)	0.103
Female	Reference		Reference	
BMI	0.79 (0.75-0.83)	< .001*	0.80 (0.76-0.84)	< .001*
AST	0.96 (0.93-0.99)	0.009*	0.99 (0.95-1.03)	0.761
ALT	0.94 (0.92-0.96)	< .001*	0.95 (0.95-1.01)	0.124

Abbreviations: LD-1: patients with cancer-associated liver disease; LD-2: patients liver disease not associated with cancer. *P value < 0.05

Several previous studies have proposed the use of specific serum markers to predict advanced liver disease. For example, hypoalbuminemia and hypergammaglobulinemia are accepted biochemical features of liver cirrhosis [10, 11]. Patients with liver cirrhosis also typically have thrombocytopenia due to the accumulation and destruction of platelets in the spleen and due to the reduced synthesis of thrombopoietin [12-14]. A recent study suggested the use of DNA-based total serum protein glycomics for the diagnosis of liver cirrhosis [15]. Another recent study [5] showed that the serum G/A ratio is elevated in hepatitis C patients with liver cirrhosis, with a remarkable OR of 31.47 (95% CI: 2.45-404; *P* = 0.008).

The limitations and complications of liver biopsy and the prospect that certain demographic characteristics and serum markers might be able to discriminate patients with cancerassociated liver diseases from those with liver diseases not associated with cancer motivated the present study. We found that age, BMI, percentage of males, G/A ratio, percentage of patients with gallstones, AST, and ALT were significantly higher in patients with liver disease (LD) relative to patients with no evidence of liver disease (non-LD). In agreement with a previous study of hepatitis C patients [5], our univariate analysis showed that the G/ A ratio was significantly higher in patients with cancer-associated liver disease. However, multivariate analysis indicated that the G/A was not independently related to the presence of cancerassociated liver disease (OR: 1.01; 95% CI: 0.99-1.02; *P* = 0.108). Instead, our multivariate analysis indicated advanced age and elevated BMI were significantly and independently associated with the presence of cancer-associated liver disease. The difference with the previous study [5] may be because that study focused on hepatitis C patients, whereas only about 1% of our LD-2 patients were positive for hepatitis C.

We used our univariate and multivariate models to construct ROC curves for G/A ratio by considering variables that were significantly associated with liver disease. The ROC curve for the univariate model, which only considered G/A as an indicator of liver disease, was very poor in predicting the presence of cancerassociated liver disease (LD-1) from liver disease not associated with cancer. In other words, the G/A ratio alone has poor sensitivity and poor specificity in the prediction of cancer-associated liver disease (Figure 2). In contrast, the ROC curve for the multivariate model yielded a much better classification system (Figure 3) Thus, for patients with liver disease, but normal levels of AST and ALT, consideration of several easily determined demographic and clinical characteristics can be used to predict the presence of cancer-associated liver disease versus liver disease not associated with cancer.

Our study is limited in that the number of subjects was not large enough to develop separate algorithms for each specific type of liver disease; our algorithm (Figure 3) only distinguishes subjects with a cancer-associated liver disease from those with liver disease not associated with cancer. Second, we did not use a validation group to test the sensitivity and specificity and of our model. Third, this was a retrospective study, so our results are more susceptible to bias and confounding than the results of prospective studies. Fourth, all of our study subjects were middleaged residents of central Taiwan who underwent health examinations, so these results may not apply to patients drawn from the general population.

Despite these limitations, our study indicates that subjects with normal AST and ALT, who were older, male, had higher BMI, lower AST, and higher ALT had significantly higher ORs for liver disease and that age and elevated BMI are significantlyo associated with the presence of cancer-associated liver disease. We can suggest several avenues for future studies. A study with a larger sample size would allow the development of an algorithm that could be used to predict specific liver diseases, much as the fibrotest is used to predict liver fibrosis [16]. We suggest that future studies consider the value of different clinical tests for predicting the presence of different classes of liver diseases, or even specific liver diseases, rather than liver disease in general.
